# Genetic Characterization and Enhanced Surveillance of Ceftriaxone-Resistant *Neisseria gonorrhoeae* Strain, Alberta, Canada, 2018

**DOI:** 10.3201/eid2509.190407

**Published:** 2019-09

**Authors:** Byron M. Berenger, Walter Demczuk, Jennifer Gratrix, Kanti Pabbaraju, Petra Smyczek, Irene Martin

**Affiliations:** University of Calgary, Calgary, Alberta, Canada (B. Berenger);; Alberta Public Laboratories, Calgary (B. Berenger, K. Pabbaraju);; Public Health Agency of Canada, Winnipeg, Manitoba, Canada (W. Demczuk, I. Martin);; Alberta Health Services, Edmonton, Alberta (J. Gratrix, P. Smyczek)

**Keywords:** *Neisseria gonorrhoeae*, azithromycin, cefixime, ceftriaxone, cephalosporin, gonorrhea, treatment failure, drug resistance, antimicrobial resistance, molecular typing, molecular epidemiology, penicillin binding protein 2, bacteria, Canada, Alberta

## Abstract

In July 2018, a case of *Neisseria gonorrhoeae* associated with ceftriaxone treatment failure was identified in Alberta, Canada. We identified the isolate and nucleic acid amplification testing (NAAT) specimen as the ceftriaxone-resistant strain multilocus sequence type 1903/NG-MAST 3435/NG-STAR 233, originally identified in Japan (FC428), with the same *penA* 60.001 mosaic allele and genetic resistance determinants. Core single-nucleotide variant (SNV) analysis identified 13 SNVs between this isolate and FC428. Culture-independent surveillance by PCR for the A311V mutation in the *penA* allele and *N. gonorrhoeae* multiantigen sequence typing directly from NAAT transport media positive for *N. gonorrhoeae* by NAAT did not detect spread of the strain. We identified multiple sequence types not previously detected in Alberta by routine surveillance. This case demonstrates the benefit of using culture-independent methods to enhance detection, public health investigations, and surveillance to address this global threat.

As incidence of *Neisseria gonorrhoeae* infection increases worldwide (*1*–*3*), sporadic cases of the ceftriaxone-resistant multilocus sequence type (MLST) 1903 (original isolate FC428 identified in Japan) continue to be detected (*4*). The emergence of extended-spectrum cephalosporin-resistant (ESCR) strains threatens the ability to treat and control the increasing number of *N. gonorrhoeae* infections.

Nucleic acid amplification tests (NAAT) are the preferred method used for diagnosis of *N. gonorrhoeae* infection because they have advantages over culture-based methods, such as enhanced sensitivity (*5*) and rapid turnaround time, and they can be automated. Because of the widespread use of NAAT, culture has been typically restricted to specific screening programs (e.g., sexually transmitted infection [STI] clinics), in cases of treatment failure, or for high-risk patients (*6*). In 2016 in Canada, ≈80% of the reported *N. gonorrhoeae* cases were diagnosed using NAAT (*7*). Historically, genomic typing and susceptibility testing required bacterial culture. Although culture is still the standard to determine antimicrobial susceptibilities, sequence typing can be performed independently from culture directly from NAAT transport media (*8*). Palmer et al. (*9*) have shown that antimicrobial resistance in *N. gonorrhoeae* is usually uniform within a given sequence type and the susceptibility pattern can be predicted for sequence types with known phenotypic susceptibilities. Genetic antimicrobial resistance markers can also be detected directly from specimens collected in NAAT transport media, including the *penA* (penicillin-binding protein 2) 60.001 allele associated with ESCR in MLST1903 (*10*).

In January 2017, a ceftriaxone-resistant *N. gonorrhoeae* strain (MIC 1 mg/L) was identified in Quebec (*11*), isolated from a 23-year-old woman whose partner had reported sexual contact during travel to China and Thailand during fall 2016. She was successfully treated with combination therapy of cefixime (800 mg orally) and azithromycin (1 g orally), followed by azithromycin (2 g orally) 13 days later. 

In July 2018, a second case of ceftriaxone-resistant *N. gonorrhoeae* infection causing urethritis was detected in Canada in the province of Alberta; the male patient probably acquired it through an anonymous sexual encounter in Alberta with a visitor from Taiwan or mainland China (*12*). Current *N. gonorrhoeae* culture protocols only capture a small portion of the *N. gonorrhoeae*-infected population and, as with the Alberta case, there can be a delay of months from initial sexual encounter to NAAT diagnosis and the determination of ESCR by culture. In the Alberta case, the infection probably occurred sometime during December 2017–February 2018; no sexual encounters were reported afterward. The *N. gonorrhoeae*–infection was diagnosed by NAAT on a urethral swab in May; the patient was treated with ceftriaxone (250 mg intramuscularly) and azithromycin (1 g orally). His urethritis recurred; he tested positive by NAAT again on a urethral swab and urine in June and was referred to the STI clinic. He sought care at the clinic in July and the initial treatment of ceftriaxone and azithromycin was repeated. Upon notification of the ESCR urethral-swab isolate, STI services treated him with gentamicin (240 mg intramuscularly) and azithromycin (2 g orally). Urethral swab culture and urine NAAT taken 4 weeks later tested negative.

This study had 2 aims. The first was to describe the microbiological investigation into this case at the local, provincial, and national levels using phenotypic antimicrobial susceptibility testing and nucleic acid–based antibiotic susceptibility determination, as well as genetic typing of the June 2018 NAAT specimen and the July 2018 Alberta isolate (designated 51742). Because anonymous sexual encounters made traditional contact investigation impossible and diagnosis was delayed, it was possible that this ceftriaxone-resistant strain had spread within Alberta. The second aim was to determine any further dissemination of the strain by expanding sequence typing and testing for the same *penA* allele using NAAT transport media specimens from clients tested in the same geographic area shortly after the case was detected.

## Methods

### Detection of *N. gonorrhoeae* and Phenotypic Antimicrobial Susceptibility Determination

NAAT-based diagnosis of *N. gonorrhoeae* infection was performed using the Aptima Combo 2 nucleic acid amplification test kit (Hologic Inc., https://www.hologic.com) for *N. gonorrhoeae* and *Chlamydia trachomatis* at Calgary Laboratory Services, Calgary, Alberta. The province of Alberta (population 4.25 million) is divided into 5 health zones (North, Edmonton, Central, Calgary, and South); Calgary Laboratory Services performs all NAAT for *N. gonorrhoeae* and *C. trachomatis* for the Calgary Zone (population 1.6 million) and South Zone (population 0.3 million) (*13*).

*N. gonorrhoeae* was cultured at the Alberta Provincial Laboratory for Public Health (ProvLab) from a specimen planted onto Thayer-Martin agar with antibiotics at the time of collection at the Calgary STI clinic. Agar plates were prepared in-house with GC agar base (BD Difco; Becton-Dickinson, https://www.bd.com). Susceptibility testing was conducted in accordance with Clinical and Laboratory Standards Institute (CLSI) M100 guidelines (*14*). Antimicrobial susceptibility was tested by gradient diffusion at ProvLab using Etest (BioMérieux, https://www.biomerieux.com), except for ertapenem, which was tested with Liofilchem (https://www.liofilchem.com) at the National Microbiology Laboratory (NML), Public Health Agency of Canada. Agar dilution was performed at the NML in accordance with CLSI M100 (*14*) for azithromycin, cefixime, ceftriaxone, erythromycin, penicillin, spectinomycin, tetracycline (Sigma-Aldrich, https://www.sigmaaldrich.com), ciprofloxacin (Bayer Healthcare, https://www.bayer.com), ertapenem (Sequoia Research, www.seqchem.com), and gentamicin (MP Biomedical, https://www.mpbio.com). Ertapenem gradient diffusion and agar dilution were done using growth supplement without cysteine. We analyzed β-lactamase production using nitrocefin (Oxoid Canada, http://www.oxoid.com).

MIC interpretations were based on CLSI M100 2019 guidelines (*15*) except for erythromycin (resistant >2 mg/L) (*16*), ertapenem (nonsusceptible >0.064 mg/L) (*17*), and gentamicin (resistant >32 mg/L) (*18*,*19*). *N. gonorrhoeae* reference cultures used as controls were ATCC49226, WHO-F, WHO-G, WHO-K, and WHO-P/WHO-U.

### Whole-Genome Sequencing of *N. gonorrhoeae* Isolate

Genomic analyses were conducted at the NML as previously described (*4*). *N. gonorrhoeae* culture was extracted by standard protocols using Lucigen MasterPure Complete DNA and RNA Extraction Kit (Mandel Scientific, http://www.mandel.ca). Multiplexed libraries were created with Nextera XT specimen preparation kits (Illumina, https://www.illumina.com). Paired-end, 300-bp indexed reads generated on the Illumina MiSeq platform yielded 567,231 reads/genome and average genome coverage of 84×. Reads were merged using FLASH (*20*) and assembled with SPAdes (*21*), yielding an average contig length of 7,081 bp; the average N50 contig length was 39,185 bp. We submitted raw whole-genome sequence (WGS) read data to the National Center for Biotechnology Information under BioProject no. PRJNA526031. We created a core single-nucleotide variation (SNV) phylogeny by mapping reads to FA1090 (GenBank accession no. NC_002946.2) using a custom Galaxy SNVPhyl workflow (*22*). We used a meta-alignment of informative core SNV positions to create a maximum-likelihood phylogenetic tree for the strains 51742, A7536, A7846, FC428, FC460, and 47707, as well as the ceftriaxone-resistant strains that the World Health Organization named as a reference panel: H041 (WHO-X), F89 (WHO-Y), and A8806 (WHO-Z) (*23*). We excluded variant calls within potential problematic regions, including repetitive regions, using MUMmer version 3.23 (http://mummer.sourceforge.net) with a minimum length of repeat region set to 150 and the minimum percent identity of repeat region set to 90%. We removed highly recombinant regions with >2 SNVs per 500 nt from the analysis (*20*). The percentage of valid and included positions in the core genome was 97.3%; 571 sites were used to generate the phylogeny. We generated the phylogenetic tree using FigTree version 1.4 (http://tree.bio.ed.ac.uk/software/figtree) (Figure).

**Figure:**
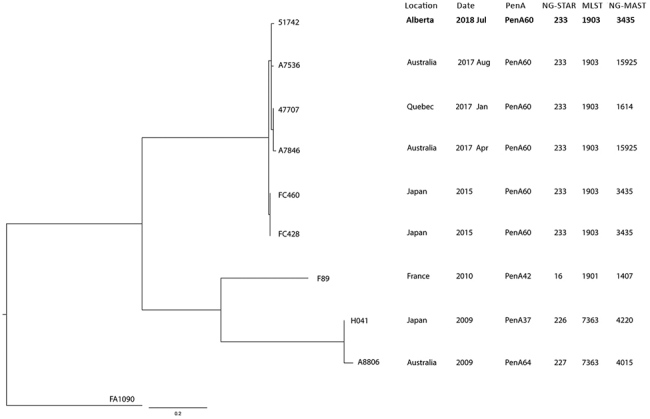
Core single-nucleotide variant (SNV) phylogenetic tree of ceftriaxone-resistant *Neisseria gonorrhoeae* identified from enhanced surveillance in Alberta, Canada (bold), and reference isolates*.* The maximum-likelihood phylogenetic tree is rooted on the reference genome of *N. gonorrhoeae* FA1090 (GenBank accession no. NC_002946.2). Scale bar represents the estimated evolutionary divergence between isolates on the basis of average genetic distance between strains (estimated number of substitutions in the sample/total number of high-quality SNVs). Strains F89 (WHO-Y), A8806 (WHO-Z), and H041 (WHO-X), A7536, 47707, A7846, FC460, and FC428 are previously reported ceftriaxone-resistant reference strains (*4*). MLST, multilocus sequence typing; NG-MAST, *N. gonorrhoeae* multiantigen sequence typing; NG-STAR, *N. gonorrhoeae* sequence typing for antimicrobial resistance.

We determined DNA sequences for *N. gonorrhoeae* multiantigen sequence typing (NG-MAST) (*24*), multilocus sequence typing (*25*), and *N. gonorrhoeae* sequence typing for antimicrobial resistance (NG-STAR) (*26*) in silico from WGS data and allocated sequence types using NG-MAST (http://www.ng-mast.net), *Neisseria* multilocus sequence typing (http://pubmlst.org/neisseria), and NG-STAR (https://ngstar.canada.ca) schemes. We determined NG-MAST of the Alberta isolate at ProvLab in accordance with Allen et al. (*27*).

### Genotyping of NAAT Specimen Collected from the Case with Treatment Failure

We referred the NAAT specimen collected in June 2018 to the NML, where DNA extraction, preparation, real-time PCR, NG-MAST, and analysis of results were performed as previously described (*28*,*29*). The NG-MAST protocol was performed with the following modifications: a nested PCR for both *porB* and *tbpB*, with *porB* using primers porF1 (5′-CAATGAAAAAATCCCTGATTG-3′) and porR1 (TTTGCAGATTAGAATTTGTGG) for the first step and primers porF2 (CTGATTGCCCTGACTTTGGCAG) and porR2 (AGAAGTGCGTTTGGAGAAGTCG) for the second step; and nested PCR for *tbpB* using primers tbpBF1 (AGGAATTGGTTTTCCGCTTT) and tbpBR1 (CGGTTTTCGCCATACCTTTA) for the first step, and *tbpB* primers by Martin et al. (*24*) for the second step. We evaluated SNV targets associated with ceftriaxone-resistant *penA* mutation (A311V), cephalosporin-decreased susceptibility (*ponA* L421P, *mtrR* 35delA, *porB* G120/A121, and *penA* mosaic allele), ciprofloxacin resistance (*gyrA* S91, D95, and *parC* D86/S87/S88), and azithromycin resistance (23S rRNA A2059G, C2611T) (*10*,*28*,*29*).

### Enhanced Surveillance of Additional NAAT Specimens

In Alberta, routine *N. gonorrhoeae* antimicrobial resistance surveillance involves sequence typing of all resistant and susceptible isolates cultured within the first 15 days of each month, collected primarily from the 3 provincial STI clinics located in Calgary (population 1.24 million), Edmonton (population 0.93 million), and Fort McMurray (population 66,653) (*30*). Because of increased rates of gonorrhea and nonavailability of cultures, all NAAT specimens from the North Zone (population 0.48 million) are typed by NG-MAST sequence typing. For further information refer to the publication by Gratrix et al. (*31*) and the Alberta Health Zone map (*13*).

We conducted NG-MAST sequence typing and testing for the *penA* A311V mutation as above from remnant clinical NAAT specimens collected during August 1–September 15, 2018, from the Calgary and South Zones in southern Alberta by the NML. Specimens collected before these dates were not tested; they were discarded in accordance with routine laboratory protocols.

## Results

Antimicrobial susceptibility by gradient diffusion at ProvLab detected an isolate resistant to ceftriaxone, cefixime, and penicillin, which was confirmed by agar dilution at the NML (Table 1). Overall, we found categorical agreement between the 2 methods, except for tetracycline, which was intermediate by gradient diffusion but resistant by agar dilution. Gentamicin was not tested by Etest due to unacceptable MIC essential agreement (<90%) compared to the agar dilution reference method (T.C. Dingle and P. Naidu, Alberta Public Laboratories, pers. comm., email, 2019 Mar 11), but the isolate was susceptible by agar dilution. Genetic resistance marker testing correlated with susceptibility phenotypes (Table 1). No specific genetic determinants of ertapenem susceptibility or nonsusceptibility have been established, but there may be overlap with mechanisms of resistance for other β-lactams (*32*). The isolate tested negative for β-lactamase production.

**Table 1:** Phenotypic and genetic antibiotic susceptibility testing for ceftriaxone-resistant *Neisseria gonorrhoeae* isolate, Alberta, Canada, 2018

Antimicrobial drug	Etest MIC, μg/mL	Agar dilution, μg/mL	Interpretation	Genetic markers/mutations identified in genome: result
Ceftriaxone	0.5–1	0.5	R	*penA*: mosaic 60.001, A311V, T483S; *mtrR* promoter: –35A deletion; *mtrR*: wild types for A39 and G45; *porB*: G120K, A121D;* porB* structure: porB1b; *ponA*: L421P
Cefixime	2	2	R	
Penicillin	4	2	R	
Ertapenem	0.064	0.125	NS	
Azithromycin	0.5–1	0.25	S	23S rRNA A2059G and C2611T: absent; *ermB*: negative; *ermC*: negative; *mtrR* promoter: –35A deletion; *mtrR*: wild types for A39 and G45
Ciprofloxacin	>32	32	R	*gyrA*: S91F and D95A; *parC*: D86, S87R, and wild type S88
Tetracycline	1	2	R†	rpsJ: rpsJ_3 (V57M); *tetM*: negative*; mtrR* promoter: –35A deletion; *mtrR*: wild types for A39 and G45
Gentamicin	ND	8	S	Genetic resistance determinants not determined
Spectinomycin	ND	16	S	
Moxifloxacin	4	ND	NA	

*Interpretations based on agar dilution. NA, breakpoint not available; ND, not done; NS, nonsusceptible; R, resistant; S, susceptible.  †Tetracycline resistance would be intermediate on the basis of Etest MIC.

Within 2 days of the isolate being identified as ESCR, sequencing of *porB* and *tbpB* at the ProvLab revealed that the isolate was NG-MAST 3435, the same sequence type as the FC428 isolate from Japan isolated in 2015 (*33*). The isolate also had the same *penA* mutations as FC 428 (A311V and T483S), providing genetic confirmation of the phenotypic resistance results. Sequence typing at NML confirmed the findings at the ProvLab and further characterized the isolate as MLST1903, NG-STAR 233, and *penA* mosaic 60.001, which were the same as other ESCR MLST1903 (*4*). WGS revealed that the isolate had <13 SNV differences from other ESCR MLST1903 isolates (Figure). The isolate was most closely related to A7536 from Australia (7 SNV) and distantly related (about 300 SNV) to other ceftriaxone-resistant isolates (France 2010 F89, Australia 2009 A8806, and Japan 2009 H041) (Figure).

In addition to the *penA* mutations*,* resistance determinants found in other ESCR MLST1903 isolates and ESCR MLST1901, including a –35A deletion in the *mtrR* promoter, the *porB* mutations G120K and A121D, *ponA* L421P, *gyrA* S91F, *gyrA* D95A, and *parC* S87R, were also found in the Alberta isolate (*4*,*34*,*35*). We detected these resistant determinants, along with *penA* A311V, by direct testing of the Alberta June 2018 NAAT specimen.

Of the 3,677 NAAT specimens tested from Calgary and South Zones during August 1–September 15, 2018, a total of 254 (from 215 cases) were positive for *N. gonorrhoeae* and 232 (from 194 cases) could be located for sequence typing (by NG-MAST) and screening for the *penA* A311V mutation. Of the 232 positive specimens, 95 (41.0%) were collected from urine, 57 (24.6%) from the pharynx, 32 (13.7%) from the vagina, 29 (12.5%) from the rectum, 11 (4.7%) from the cervix, and 8 (3.5%) from the urethra. All specimens tested negative for the *penA* A311V mutation*.* We were able to determine the ST from 173 specimens (74.6% of specimens), each representing 1 case (89% of cases): 90 urine, 28 vaginal, 24 rectal, 14 pharyngeal, 9 cervical, and 8 urethral specimens. Of the 232 positive specimens, 59 (25.4%) were nontypeable because of either poor sequence quality or no amplicon for one or both targets; 8 had a *porB* sequence only and 38 *tbpB* only. Among the 173 specimens sequence typed, we detected 53 different NG-MAST; the most prevalent STs (>10 specimens) were ST8890 (34; 19.7%), ST5441 (17; 9.8%), and ST16065 (12; 6.9%) (Table 2). We did not detect NG-MAST-3435 in these specimens or in any other specimen or isolate typed as a part of routine testing in Alberta as of June 30, 2019. We did not have culture data available for 30 sequence types (56.6%) to predict susceptibility. 

**Table 2:** Test results and antimicrobial resistance pattern predictions of sequence types identified from enhanced surveillance of *Neisseria gonorrhoeae* samples, Alberta, Canada*

Sequence type by NG-MAST	No. specimens (% of all specimens), n = 232†	No. cases, n = 194†	Antimicrobial resistance pattern prediction‡	No. specimens with specified AMR/no. cultures identified with ST§
Nontypeable	59 (25.4)	35	NA	
ST8890	34 (14.7)	33	TetR	43/45
ST5441	17 (7.3)	17	Susceptible	66/79
ST16065	12 (5.2)	9	CipR/TetR	63/63
ST14788	7 (3.0)	6	Susceptible	21/26
ST5985	7 (3.0)	7	TRNG	649/730
ST11086	6 (2.6)	5	CipR/EryR/PenR/TetR	30/30
ST12302	6 (2.6)	6	CipR/EryR/TetR/often AziR	1,198/1,211 (CipR/EryR/TetR); 687/1,211 (are also AziR)
ST4637	6 (2.6)	5	Susceptible	17/20
ST8288	6 (2.6)	6	Penicillinase-producing/CipR	12/14
ST1387§	4 (1.7)	4	No culture available	0
ST16288	4 (1.7)	4	CipR/EryR	26/26
ST16972	4 (1.7)	4	Susceptible	4/5
ST17029	4 (1.7)	4	No culture available	0
ST18135	4 (1.7)	2	No culture available	0
ST4186	4 (1.7)	4	Susceptible	4/4
ST11461	3 (1.3)	3	TRNG	161/166
ST13489	3 (1.3)	3	Susceptible	1/1
ST16211	3 (1.3)	2	EryR/TetR	2/2
ST14698	2 (0.86)	2	AziR/CipR/EryR/TetR	142/208
ST16825§	2 (0.86)	1	No culture available	0
ST18126§	2 (0.86)	2	No culture available	0
ST18154	2 (0.86)	2	No culture available	0

*N. gonorrhoeae infection was diagnosed by the Aptima Combo 2 nucleic acid amplification test kit (Hologic Inc., https://www.hologic.com). AziR, azithromycin resistant; CipR, ciprofloxacin resistant; EryR, erythromycin resistant; NG-MAST, *N. gonorrhoeae* multiantigen sequence typing; ST, sequence type; TetR, tetracycline-resistant; TRNG, TetR *N. gonorrhoeae* (high-level plasmid mediated). †Sequence types detected in only 1 specimen and resistance patterns (if known) are as follows and represent individual cases unless noted as an additional site: ST10451 (PenR/CipR/EryR/TetR); ST11691 (CipR/EryR/TetR and half are AziR); ST11933 (susceptible or TetR); ST-16161 (TetR); ST16595 (TRNG); ST18146; ST18156; ST3935 (EryR/TetR, sometimes PenR); ST9120. Sequence types detected in only 1 specimen that had not previously been detected in Alberta are ST14797; ST17196; ST18057; ST18134; ST18136; ST18137; ST18138; ST18155 (additional site); ST18157; ST18158; ST18159; ST18160; ST18161; ST18162; ST18340 (additional site); ST18341; ST18342; ST18343; ST18344; ST18345 (additional site); ST5308 (XDR: AzR/CeDS/CipR/PenR/TetR/EryR); ST6339.  ‡Based on data from the Canadian national gonococcal collection, which included 7,498 gonococcal cultures with sequence typing and phenotypic susceptibility results collected 2016–2018.  §Sequence type had not been previously detected in Alberta.

The enhanced surveillance during August 1–September 15, 2018, resulted in successful sequence typing of 89% of 215 cases in the Calgary and South Zones and 42.5% of 703 cases in all Alberta health zones. In comparison, the sequence typing success rate for December 1, 2017–July 31, 2018, was 18.5% (139/748) of *N. gonorrhoeae* infection cases in Calgary and South Zones and 18.5% (566/2,807) of cases in all of Alberta. 

Of the 194 cases included in the enhanced surveillance specimens, 22 (11.3%) also had a culture-positive isolate with a sequence type identified through routine surveillance. In 11/22 cases, the ST from the NAAT matched the isolate. In 10 of 26 NAAT specimens from the 22 cases with an isolate available, the NAAT ST was nontypeable, and in 1 case, the ST isolate and NAAT (both collected on the same day from the same pharynx sample) differed by 1 nucleotide, indicating that the infecting strain had mutated. Twenty-three cases had multiple sites of infection, and 6 of these had distinct strains at each site, determined by either different STs (n = 3) or *porB* (n = 2) or *tbpB* (n = 1) alleles. We found no evidence of mixed infections at the same site.

The additional surveillance of NAAT specimens in the Calgary and South Zones for 1.5 months identified 26 new NG-MAST for Alberta. Most of these new ST (n = 24, 92.3%) represented individual cases. ST1387 was new to Alberta with 4 cases and ST18126 with 2 cases (Table 2). 

## Discussion

Continued detection and control of ESCR globally requires rapid detection of cases, coordinated public health interventions, and enhanced surveillance for antimicrobial resistance. Critical to the detection and prevention of further cases from the laboratory standpoint is the maintenance of culture and susceptibility testing along with rapid molecular confirmation of resistance and determination of sequence type. Because diagnosis of *N. gonorrhoeae* infections by NAAT is far more widespread than by culture, NAAT specimens can be used to detect ESCR in the population by culture-independent methods. The detection of the second case of ceftriaxone resistance in Canada highlights the use of these methods to facilitate public health investigation in a timely manner. Our findings show that current surveillance activities fail to conduct sequence typing in 80% of cases. Laboratory detection must, of course, be accompanied by appropriate clinical case management and public health follow-up, including partner notification and test of cure for treatment of drug-resistant or treatment failure cases.

In the Alberta ESCR case, we could not exclude the possibility of further spread of the ESCR because of the patient’s anonymous sexual encounters and the long delay in diagnosis. Alberta and other provinces in Canada currently rely on susceptibility testing in select populations (e.g., STI clinic patients) to detect *N. gonorrhoeae* resistance. This surveillance covers only 22% of the cases in Alberta (*31*); alternative approaches to susceptibility testing, such as screening for resistance markers and typing of NAAT specimens, are required for greater representation of the population. 

Upon sequence typing specimens for 6 weeks after detection of the ESCR case, we found 26 STs that were previously not detected in Alberta. Although no ceftriaxone-resistant specimens were detected, 1 was identified as NG-MAST-5308, which had not been previously detected in Alberta and has been associated with multidrug resistance and decreased susceptibility to cephalosporins (I. Martin, unpub. data). ST5308 has been circulating in a neighboring province and, upon further investigation, we found that the case-patient was a resident of that province.

In line with World Health Organization recommendations (*36*), our study supported the value of enhanced culture-independent surveillance of NAAT specimens after detection of an ESCR isolate by sequence typing and PCR for resistance markers such as *penA* mutations. In our study, the ad hoc rapid population surveillance indicated that the strain was not circulating in our population by increasing the percentage of *N. gonorrhoeae* cases sequence typed in the Calgary and South Zones from 18.5% to 89%. The appropriate follow-up period for enhanced surveillance should take into account local baseline surveillance practices and the risk of transmission into the local population. Our risk assessment took into account the clinical and sexual history, number of sexual encounters, location of the sexual encounters, ability to reach case-contacts, and resource availability to run culture-independent surveillance. In our study, the risk for transmission was determined to be low based on the patient’s sexual history, and we chose not to do any further enhanced surveillance.

Sequence typing and NAAT-based detection of genetic resistance determinants can act as a surrogate for culture (*8*,*9*); however, there are limitations. First, the susceptibility pattern of the sequence type needs to be known (only 44% were in our study), and the correlation between sequence type and susceptibility pattern needs to be well documented. The detection of genetic resistance markers by molecular techniques can also be challenging when multiple resistance markers can contribute to a single phenotype or the markers are unknown (as is the case with ertapenem [*32*)]). Fortunately, in the case of NG-MAST 3435, the specific *penA* allele is known and the method for nucleic acid–based detection of this allele has been published (*10*). Advances in streamlining the methods for sequence typing and resistance marker detection from NAAT specimens are also needed because the current methods are labor intensive and costly. Of note, performing nucleic acid–based surveillance requires the availability of specimens. In our case, the laboratory stored positive specimens routinely for 1 month after collection, but upon detection of the ESCR case, this time was changed to 2 months. Longer storage times of positive samples would improve surveillance of NAAT specimens collected before an ESCR isolate is detected by culture. We were also able to get specimens easily, because CT/GC NAAT is centralized to 1 site in southern Alberta.

Additional phenotypic susceptibility testing of our isolate was challenging within the province because agar dilution was not available and published evaluations comparing the accuracy of gradient diffusion methods for alternate antibiotics, especially aminoglycosides, are lacking. In the absence of accurate susceptibility testing for gentamicin, STI services treated the Alberta case empirically based on Canada’s STI guidelines. The previously unreported MIC of the Quebec 2017 isolate (47707) was determined by agar dilution at NML to be 0.125 µg/mL; our results would indicate that NG-MAST 3435/MLST1903 isolates should be considered nonsusceptible to ertapenem. Ertapenem has also been used as a treatment option for an ESCR case in the United Kingdom (*37*), but most ESCR publications have not reported the MICs for ertapenem, other than the United Kingdom case (0.032 µg/mL; susceptible) (*37*) and a case in France (0.004 µg/mL; susceptible) for which only Etest results were reported (*34*). We recommend that future descriptions of ESCR include MICs for all antimicrobial drugs that are considered treatment options by both gradient diffusion and agar dilution.

To curtail the international spread of ESCR strains, proactive surveillance by molecular techniques may be necessary to detect cases, especially where culture is not available. Jurisdictions should also consider increasing the number of cases for which culture is available. Once a case is found, rapid molecular confirmation of ESCR strains to determine the possible origin and confirm the phenotypic results is imperative to aid public health authorities. Consideration of optimal surveillance strategies needs to be part of a global effort that encompasses continued international surveillance; open and complete data sharing; and enhancement of laboratory, treatment, and public health support to resource-limited areas of the world.
